# Body Adiposity Index and Cardiovascular Health Risk Factors in Caucasians: A Comparison with the Body Mass Index and Others

**DOI:** 10.1371/journal.pone.0063999

**Published:** 2013-05-29

**Authors:** Miquel Bennasar-Veny, Angel A. Lopez-Gonzalez, Pedro Tauler, Mey L. Cespedes, Teofila Vicente-Herrero, Aina Yañez, Matias Tomas-Salva, Antoni Aguilo

**Affiliations:** 1 Research Group on Evidence, Lifestyles and Health, Universitat Illes Balears, Palma, Spain; 2 Prevention of Occupational Risks in Health Services, GESMA, Balearic Islands Health Service, Palma, Spain; 3 Fundamental Biology and Health Sciences Department, Universitat Illes Balears, Palma, Spain; 4 Prevention of Occupational Risks, Correos, Valencia, Spain; 5 Prevention of Occupational Risks, Balearic Islands Government, Palma, Spain; University College London, United Kingdom

## Abstract

**Background:**

Several studies have shown a relation between the adipose tissue accumulation and a higher risk for developing metabolic and cardiovascular diseases. Thus, body fat content and, mainly, the fat distribution or adiposity could be considered as important indicators of health risk. In spite of presenting several limitations, BMI is the most widely used and accepted index for classifying overweight and obesity. The aim of the study was to evaluate the correlations between Body Adiposity Index (BAI), BMI and other adiposity indexes such as WC, WHR and WHtR with cardiovascular and metabolic risk factors. Furthermore, the behavior of BAI and BMI regarding the ability to discriminate overweight or obese individuals was also analyzed.

**Research Methodology/Principal Findings:**

A cross-sectional study was conducted in Spanish Caucasian adult workers. Participants in the study (29.214 men and 21.040 women, aged 20–68 years) were systematically selected during their work health periodic examinations. BAI, BMI, WHR, WHtR, body weight, hip and waist circumference (WC) as well as systolic and diastolic blood pressure were measured. Serum levels of high density lipoprotein cholesterol (HDL-C), low density lipoprotein cholesterol (LDL-C), triglycerides (TG) and glucose were also determined. Results of the study indicated that BAI was less correlated with cardiovascular risk factors and metabolic risk factors than other adiposity indexes (BMI, WC and WHtR). The best correlations were found for WHtR. In addition, the BAI presented lower discriminatory capacity than BMI for diagnosing metabolic syndrome (MS) using both IDF and ATP III criteria. A different behavior of the BAI in men and women when considering the ability to discriminate overweight or obese individuals was also observed.

**Conclusions:**

The adiposity indexes that include the waist circumference (WHtR and WC) may be better candidates than BAI and BMI to evaluate metabolic and cardiovascular risk in both clinical practice and research.

## Introduction

Obesity is a chronic and complex disease which is defined as an excess of body fat. Due to continuous increase in prevalence in adults, adolescents and children, obesity has become one of the most important public health problems. The increase in prevalence of obesity involves an increase in the prevalence of several obesity-related comorbidities [1]–[3]. Among others, adiposity is supposed to be the physiological characteristic of obese and overweight individuals, which puts such individuals at-risk for cardiovascular disease [4]. In fact, the relationship between overall adiposity and risk for cardiovascular disease is well documented [5], [6]. Furthermore, several studies, including the Framingham heart study [7], shows the relation between the adipose tissue accumulation and the incidence of adverse metabolic events and, also, with a higher risk for developing metabolic diseases [8]–[10]. In fact, in Spain Framingham equation has been adjusted to allow its utilisation as an effective predictor for cardiovascular risk [11], [12].

Thus, body fat content and, mainly, the fat distribution or adiposity could be considered as important indicators of health risk. Several techniques have been developed for assessing and/or determining body fat or adiposity. Among others, these methodologies include the body mass index (BMI), waist circumference (WC), waist-to-hip ratio (WHR), waist-to-height ratio (WHtR), skinfold thickness, dual-energy X-ray absorption (DXA) and hydrostatic densitometry. The BMI, an index of relative weight, is the most widely used and accepted index for classifying overweight and obesity in clinical practice, providing a simple approach to characterize obesity in individuals [13]. However, BMI presents some important and well documented limitations, such as: a different behaviour in men and women, limited usefulness in children and athletes, differences between ethnic groups and especially in determining the composition and distribution of body fat, which can represent a limitation in epidemiological studies or clinical practice. Among other errors, the above indicated limitations could lead to classify individuals with high muscle mass as overweight or obese. On the other hand, subjects with BMI in the normal range may have a high percentage of fat [14]–[19].

Bergman et al. suggested an alternative index, the body adiposity index (BAI) based on the measurements of hip circumference and height. This index showed a high correlation with body fat measured using DXA (r = 0.85, P<0.001). In their study, conducted only in two U.S. ethnic populations, African Americans and Mexican Americans, Bergman et al. found that this correlation was higher than the one between BMI and body fat measured using DXA when men and women were considered together [4]. The authors concluded that the BAI is a useful predictor of obesity and suggested that involves more simple measurements because weight is not needed. However, it has been recently suggested that BAI does not overcome the limitations of BMI, being the fact that weight is not needed the only advantage of BAI over BMI [20].

The metabolic syndrome (MS) is a set of interrelated risk factors such as hypertension, dyslipidemia, obesity and high blood glucose [21], [22]. The clustering of cardiovascular disease risk factors that typifies the metabolic syndrome appears to confer substantial additional cardiovascular risk above the addition of the risk associated with each individual factor [23]. Insulin resistance together with central/abdominal or visceral obesity have been proposed as the key factors in the development of the MS [24]–[26].

Several authors have tested the correlations between the indexes of adiposity and several health outcomes [5]–[7], an issue that the original authors of BAI did not address [4]. A recent study has reported that BAI could be less useful than BMI when the metabolic health risk is evaluated [27]. Furthermore, this study suggested that WC and WHR may be even better candidates than BMI or BAI as simple (only tape measurements are required) and practical indicators of cardiovascular health risk [27]. Taking into account these observations, the aim of the present study was to analyse, in a large population, the correlations between BAI, BMI and other adiposity indexes (WC, WHR and WHtR) with cardiovascular and metabolic risk factors. Furthermore, the behavior of the BAI and BMI regarding the ability to discriminate overweight or obese individuals was also analyzed.

## Materials and Methods

### Subjects and Study Protocol

A cross-sectional study with Caucasian adult workers (ages, 20–68 years) was performed. All subjects were from Mallorca (Spain) and belong to different productive sectors. Participants in the study were systematic selected during their work health periodic examinations between January and December 2011. Every day each worker was assigned a number and half of the examined workers were randomly selected using a random number table. Thus, from a total population of 130,487 workers, 65,200 of them were invited to participate in the study. 14,946 (22.9%) refused to participate, being the final number of participants 50,254 (77.1%), with 21,040 women (41.9%) and 29,214 men (58.1%). The mean age of participants in the study was 39.90 years (SD ±10.33). All participants were informed of the purpose of this study before they provided written informed consent to participate. Following the current legislation, members of the Health and Safety Committees were informed as well. The study protocol was in accordance with the Declaration of Helsinki and was approved by the Institutional Review Board of the Mallorca Health Management (GESMA). After acceptance, a complete medical history, including family and personal history, was recorded. The following inclusion criteria were considered: age between 18 and 70 years (working age population), agreement to participate in the study and to be gainfully employed. Subjects who did not meet any of the inclusion criteria and those who refused to participate were excluded from the study.

### Measurements and Calculations

All anthropometric measurements were made in the morning, after an overnight fast, at the same time (9 a.m.), and according to the recommendations of the International Standards for Anthropometric Assessment (ISAK) [28]. Furthermore, all measurements were performed by well trained technicians or researchers to minimize coefficients of variation. Each measurement was made three times and the average value was calculated. Weight and height were determined according to recommended techniques mentioned above. Body weight was measured to the nearest 0.1 kg using an electronic scale (Seca 700 scale, Seca gmbh, Hamburg). Height was measured to the nearest 0.5 cm using a stadiometer (Seca 220 (CM) Telescopic Height Rod for Column Scales, Seca gmbh, Hamburg). BMI was calculated as weight (kg) divided by height (m) squared (kg/m^2^). Criteria used to define overweight were the ones of the World Health Organization (WHO) [29], which considers obesity when BMI ≥30 kg/m^2^. Abdominal waist and hip circumferences were measured using a flexible steel tape (Lufkin Executive Thinline W 606). The plane of the tape was perpendicular to the long axis of the body and parallel to the floor. Waist circumference was measured at the level of the umbilicus and the superior iliac crest. The measurement was made at the end of a normal expiration while the subject stood upright, with feet together and arms hanging freely at the sides. Hip circumference was measured over non-restrictive underwear or light-weight shorts at the level of the maximum extension of the buttocks posteriorly in a horizontal plane, without compressing the skin.

BAI was calculated using the equation ((hip circumference)/((height)^1.5^)-18), which refers to Bergman et al. [4]. Values obtained were classified in low, normal, high and very high according to criteria established by Gallagher et al. for white population [30].

Waist circumference (WC) and hip circumference (HC) were measured using a tapeline at the level midway between the lateral lower rib margin and iliac crest as well as at the levels of trochanters. WHR was calculated as WC divided by HC. WHtR was calculated by dividing WC by height in cm [31].

Venous blood samples were taken from the antecubital vein with suitable vacutainers without anticoagulant to obtain serum. Blood samples were taken following a 12 h overnight fast. Participants were seated at rest for at least 15 minutes before blood samples were taken. Serum was obtained after centrifugation (15 min, 1,000 g, 4°C) of blood samples. Serum was stored at −20°C and analysis were performed within 3 days. Concentrations of glucose, cholesterol and triglycerides were measured in serum by standard procedures used in clinical biochemistry laboratory using a clinical system Beckman Coulter SYNCHRON CX®9 PRO (Beckman Coulter, Brea, CA, USA).

Blood pressure was determined after a resting period of 10 minutes in the supine position using an automatic and calibrated sphygmomanometer OMRON M3 (OMRON Healthcare Europe, Spain). As indicated for the anthropometrical measures, blood pressure was measured three times with a one-minute gap between each measurement and an average value was calculated.

The presence of MS was ascertained by using the criterion suggested by the National Cholesterol Educational Program Adult Treatment Panel III (NCEP ATP III) and International Diabetes Federation (IDF). Characteristics included in the ATP III criterion are: abdominal obesity (given as waist circumference, men >102 cm and women >88 cm), triglycerides ≥150 mg/dL, HDL-cholesterol <40 mg/dL in men and <50 mg/dL in women, blood pressure ≥130/85 mm Hg, fasting glucose ≥100 mg/dL. When 3 of 5 of the listed characteristics are present a diagnosis of metabolic syndrome can be made [32]. Characteristics included in the IDF criteria are: central obesity (defined as waist circumference, men ≥94 cm and women ≥80 cm. If BMI is >30 kg/m^2^, central obesity can be assumed and waist circumference does not need to be measured), triglycerides ≥150 mg/dL or specific treatment for this lipid abnormality, HDL-cholesterol <40 mg/dL in men and <50 mg/dL in women, blood pressure ≥130/85 mm Hg or treatment of previously diagnosed hypertension, fasting plasma glucose >100 mg/dL or previously diagnosed type 2 diabetes. When central obesity plus two of the four previous criteria are met, a diagnosis of metabolic syndrome can be made [33]. In order to determine the cardiovascular risk, two different equations were used: 1) the Framingham risk equation [34] and 2) the REGICOR method, which supposes an adaptation to the risk factor prevalence and event characteristics of the population in Spanish population [11].

### Statistical Analyses

All the data were tested for their normal distribution (Kolmogorov–Smirnov test). Results are expressed as means and standard deviations (SD) and, when required, in percentages. Student t test for unpaired data was used to evaluate differences in anthropometric and biochemical characteristics between genders (Table 1). The existence of significant bivariate correlations between parameters such as BAI, BMI, WC, WHR, WHtR and biochemical parameters, cardiovascular risk indexes (Framingham and REGICOR) and metabolic risk factors was ascertained by determining Pearson or Spearman correlation coefficients. The statistical method of ROC curves (Receiver operating characteristic curves were used to determine BMI and BAI discriminatory capacity of metabolic syndrome). Cutoff values were derived mathematically from the ROC curves. Statistical analysis was carried out using IBM SPSS Statistics 20.0 software (SPSS/IBM, Chicago, IL, USA). Significance was accepted at p<0.05.

**Table 1:** Anthropometric characteristics, biochemical circulating parameters and Cardiovascular Risk (CVR) of participants in the study (n = 50,254).

Characteristics^1^	Total (n = 50,254)	Men (n = 29,214)	Women (n = 21,040)	*P* value^2^
Age (years)	39.90±10.33	40.30±10.51	39.35±10.05	<0.001
Weight (kg)	75.02±14.92	80.76±13.34	67.04±13.22	<0.001
Height (cm)	169.05±8.80	173.90±6.96	162.30±6.30	<0.001
BMI (kg/m^2^)	26.20±4.60	26.70±4.14	25.50±5.09	<0.001
BAI (kg/m^2^)	27.45±4.36	26.32±3.64	29.02±4.77	<0.001
Waist circumference (cm)	83.64±11.59	88.77±9.65	76.53±10.23	<0.001
Hip circumference (cm)	99.63±8.67	101.50±8.13	97.03±8.72	<0.001
WHR	0.84±0.09	0.88±0.08	0.79±0.08	<0.001
WHtR	0.49±0.06	0.51±0.06	0.47±0.07	<0.001
HDL-C (mg/dl)	52.46±8.74	50.54±7.70	55.12±9.38	<0.001
LDL-C (mg/dl)	120.76±37.15	121.14±37.26	120.23±36.99	<0.001
Triglycerides (mg/dl)	109.09±75.86	123.64±88.60	88.89±46.29	<0.001
Glucose (mg/dl)	88.22±18.78	90.16±20.73	85.52±15.27	<0.001
Systolic BP (mmHg)	121.03±16.25	125.27±15.67	115.15±15.17	<0.001
Diastolic BP (mmHg)	73.91±10.96	75.99±10.85	71.02±10.45	<0.001
CVR REGICOR	2.38±2.06	2.79±2.30	1.80±1.49	<0.001
CVR Framingham	5.65±5.72	7.29±6.35	3.36±3.61	<0.001
MS ATP III (%)	8.50	10.50	5.60	<0.001
MS IDF (%)	8.10	11.40	3.40	<0.001
Current smoker (%)	34.80	36.70	32.10	<0.001

BMI, body mass index; BAI, body adiposity index; WHR, waist-to-hip ratio; WHtR, waist-to-height ratio; HDL-C, high-density lipoprotein-cholesterol; LDL-C, low-density lipoprotein-cholesterol; Systolic BP, systolic blood pressure; Diastolic BP, diastolic blood pressure; CVR REGICOR, cardiovascular risk REGICOR; CVR Framingham, cardiovascular risk Framingham; MS ATPIII, metabolic syndrome adult treatment panel III; MS IDF, metabolic syndrome international diabetes federation.

1 Data are expressed as means ± SD.

2 Statistical significance was estimated by independent t-test, Mann-Whitney U test or χ^2^.

## Results

Age and anthropometrical characteristics of the participants in the study as a whole and categorized by gender are shown in Table 1. Significant differences between men and women were found in all parameters analysed with higher values of weight, height, BMI, waist and hip circumferences, WHR and WHtR in men than in women. On the other hand, women had higher BAI value than men. Blood parameters, cardiovascular risk, metabolic syndrome incidence as well as smoking prevalence are also shown in Table 1. Women presented higher HDL levels and men had higher LDL, triglycerides and glucose serum concentrations. Regarding blood pressure, men presented higher values of both systolic and diastolic blood pressure. Cardiovascular risk was high in men using both REGICOR and Framingham equations. Prevalence of metabolic syndrome was much higher in men than in women using both the ATP III (1,88 times higher) and the IDF criteria (3,35 times higher). Finally, among participants in the study the percentage of smokers was higher in men than in women.

**Figure 1:**
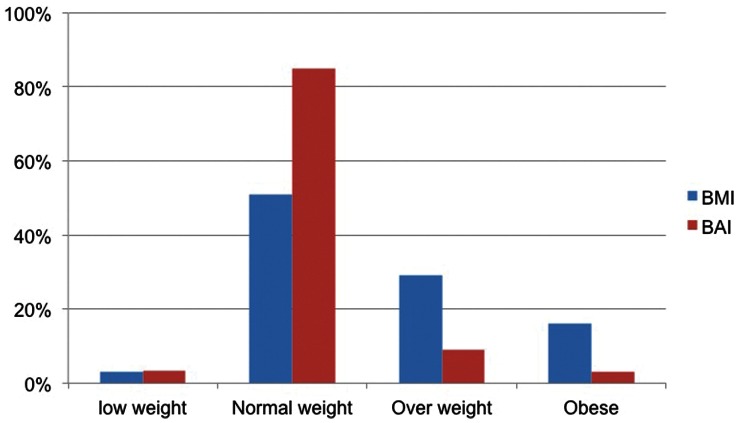
Distribution (%) of women in BMI and BAI categories.

**Figure 2:**
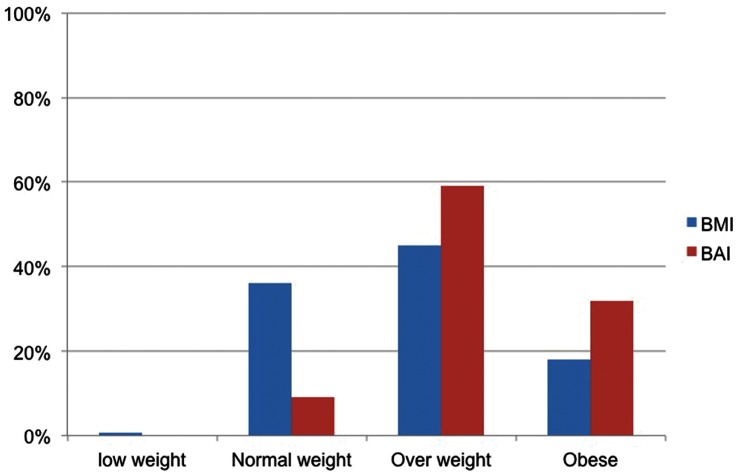
Distribution (%) of men in BMI and BAI categories.

Obesity prevalence in the whole sample was 17.7% (16.9% in women and 18.3% in men) using BMI classification (BMI ≥30 kg/m^2^) and 19.7% (2.7% in women and 32.0% in men) using BAI classification. Figures 1 and 2 show the comparison between the prevalence of overweight and obesity in men and women using BMI and BAI categories. Using BAI higher percentages of men are classified as overweight and obese than using BMI. However, in women, higher percentages are classified as overweight and obese using BMI than using BAI.

Table 2 shows the coefficients of bivariate correlations between anthropometric measures and cardiovascular risk factors. HDL and triglycerides showed the highest correlation with WC and the lowest with BAI. In general, correlations for LDL were the lowest, being the highest the one with BMI. Glucose presented the highest correlation with WHtR, being similar to the one with BMI, and the lowest with the BAI. Regarding blood pressure measurements, correlations with BAI and WHR were clearly lower than correlations with BMI, WC and WHtR. A similar pattern was observed when correlations with REGICOR and Framingham values were analysed. Correlations with BAI were lower than the other ones. It is noteworthy that both REGICOR and Framingham showed the highest correlations with WHtR.

**Table 2:** Correlations between anthropometric measures and cardiovascular risk factors.

	BMI	WC	WHR	WHtR	BAI
Age	0.263*	0.194*	0.162*	0.283*	0.245*
Height	−0.046*	0.396*	0.226*	0.021*	−0.504*
Weight	0.844*	0.752*	0.298*	0.616*	0.358*
HDL-C	−0.249*	−0.268*	−0.196*	−0.247*	−0.081*
LDL-C	0.133*	0.081*	0.076*	0.124*	0.104*
Triglycerides	0.270*	0.387*	0.311*	0.379*	0.122*
Glucose	0.215*	0.205*	0.140*	0.220*	0.131*
Systolic BP	0.369*	0.394*	0.260*	0.373*	0.170*
Diastolic BP	0.385*	0.372*	0.223*	0.371*	0.222*
REGICOR	0.271*	0.305*	0.279*	0.384*	0.158*
Framingham	0.284*	0.360*	0.354*	0.432*	0.125*

The level of significance was *p<0.01. The index associated with the highest correlative strength to the variable in the same row is highlighted.

Pearson or Spearman correlation coefficient.

**Figure 3:**
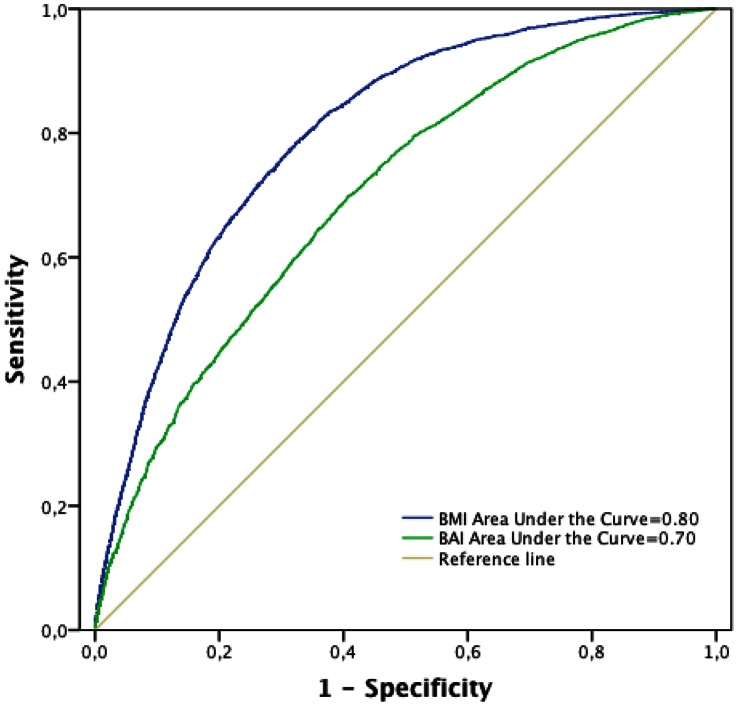
ROC curve analysis for anthropometric measures a metabolic syndrome (ATP III criteria).

**Figure 4:**
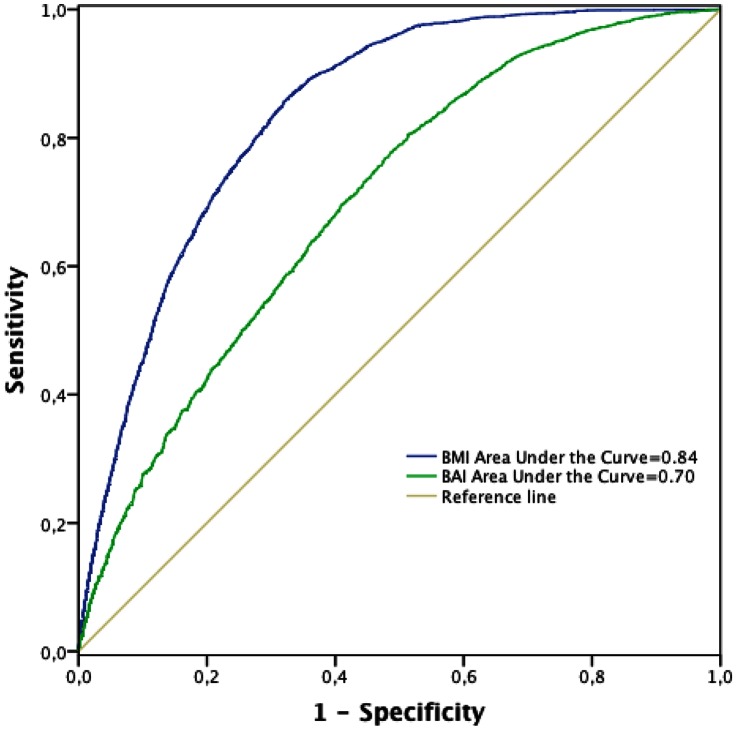
ROC curve analysis for anthropometric measures a metabolic syndrome (IDF criteria).

Figures 3 and 4 show the ROC curves for BMI and BAI respect to the presence of MS determined using both the ATP III and the IDF criteria. Taking into account the areas under the curves, BMI showed higher discriminatory capacity than BAI to determine the presence of MS measured using both ATP III and IDF criteria. When the ATP III criteria were used (Figure 3), the cutoff point value of 27.16 for the BMI provided a sensitivity of 78% (95% CI: 77%–80%) and specificity of 68% (95% CI: 67%–68%). The ROC curve for BAI was also obtained. The cutoff point was 27.47 and, considering this cutoff point, the sensitivity was 70% (95% CI: 68%–71%) and specificity was 59% (95% CI: 59%–60%). When the IDF criteria were used (Figure 4), the cutoff point value of 27.15 for the BMI provided a sensitivity of 86% (95% CI: 84%–87%) and a specificity of 68% (95% CI: 68%–68%). When the ROC curve for BAI was also analysed, the cutoff point of 26.76 provided a sensitivity of 78% (95% CI: 76%–78%) and the specificity was 51% (95% CI: 51%–52%).

## Discussion

To the best of our knowledge this is one of the first studies focused on Caucasian individuals that evaluates the applicability of BAI as a method to determine metabolic and cardiovascular risk factors in this population, comparing these values with the ones of BMI and, also, with measures obtained using indexes such as WC, WHR and WHtR. The main finding of the present study is that BAI, in spite of being a good adiposity predictor, does not overcome the limitations of BMI and the other indexes analyzed.

The predictor indexes for body composition and risk factors are widely used in the clinical practice. It has been shown that, because of the metabolic differences between the abdominal fat (especially visceral) and the gluteus fat, the body fat distribution is a stronger cardiovascular risk predictor than the obesity or the overall amount of adipose tissue [4], [27], [35], [36]. Furthermore, there is evidence that cardiovascular risk increases with increased visceral adipose tissue [37], [38]. In fact, it has been shown that visceral adipose tissue is closely associated with coronary disease [31] and is believed to be a major contributor for developing cardiovascular diseases and type 2 diabetes. In agreement with this last observation, an increased risk for developing both diseases was found among patients suffering from metabolic syndrome [39]. Previous studies had shown that BMI was not a good indicator of cardiovascular risk, particularly when it is used as the only indicator, mainly because it is not able to differentiate between adipose and muscle tissue [31]. Furthermore, BMI is not able to differentiate between fat compartments, an essential issue because visceral adipose tissue has been shown to be more associated with cardiovascular risk than subcutaneous adipose tissue [31], [36], [40]. Several researchers have concluded that abdominal obesity, usually evaluated by the WC, is more strongly associated with cardiovascular risk factor levels than BMI [15], [41]. Results obtained in the present study are in agreement with these observations because we reported that correlations between WC, WHR and WHtR and cardiovascular risk factors are better than the one with BMI, which is also in agreement with results obtained by Snijder et al. showing that [27].

We also aimed to evaluate the usefulness of BAI as a cardiovascular health risk marker. Since the BAI, as BMI, does not consider the waist circumference, it could be expected that the correlations between CVR and the BAI do not improve the ones with other adiposity indexes. In fact, Freedman et al. found that the BAI was less associated with cardiovascular risk factors than BMI or WC [15]. Results from the present study show that the correlation between BAI and CVR is not stronger than the ones with more simple indexes such as WHtR, WHR, WC and, also, with BMI. Thus, the utilization of WHtR or the WC could be recommended as simples and practical indicators for assessing cardiovascular risk. It is noteworthy that, in the present study (data not shown) the categorization of participants in the study by BMI categories (normal weight, over weight and obesity) results regarding the correlations between the BAI and the indicators of cardiovascular risk did not improve when compared with the other ones. Longitudinal studies focused on the abdominal obesity have reported inconsistent results, with some of them showing that WC could be better than BMI as cardiovascular disease predictor [31], [36] although other studies reported similar results for both WC and BMI [9], [31]. On the other hand, it has been shown that WHtR, the best indicator of visceral adipose tissue, is the best predictor of cardiovascular risk [31]. In the present study, WHtR has shown the highest correlations with the indexes obtained by both the Framingham and the REGICOR equations. Thus, and in agreement with previous studies, we could suggest the utilization of WHtR as the best adiposity index in relation to the cardiovascular health risk. However, the usefulness of indexes such as WC and WHR should also be taken into account. Both of them have shown high correlations, higher than the ones of BMI and BAI, and present the advantage over WHtR that only tape measurements are required. All the associations evaluated in the present study should be confirmed with more accurate estimations of body fat such as the ones obtained using DXA.

Regarding MS, using both ATP III and IDF definitions, BMI presented higher discriminatory capacity (higher area under the curve) than the BAI. The BAI supposes a new approach in order to determine the adiposity. The ROC curves approach was used to ascertain whether higher BAI values were associated with metabolic syndrome. Using this methodology, with BAI as continuous variable and optimizing the cut-off points, it is observed that sensitivity and specificity in the categorization of the MS are moderate and lower than the ones obtained using the BMI. Taking into account these results it is unlikely that the BAI supposes an useful predictor for high MS risk.

In agreement with our previous study [20], a different behavior of the BAI in men and women when considering the ability to discriminate overweight or obese individuals has been observed in the present study. In our previous study, we reported that the BAI overestimates fat levels in men and underestimates these fat levels in women. In the present study, when BAI and BMI categorizations were compared, a trend to classify a higher percentage of men as overweight or obese was observed. On the other hand, in women and using the cut-off points recommended for BAI, a significant trend to classify most of the women as normal weight (over 80% of women participating in the study) was observed. This inaccurate classification of body fat mass could lead to obesity risk. These observations should be taken into account when BMI or BAI are considered in order to choose the more adequate adiposity index. It is noteworthy that in the present study, the percentage of subjects with BMI≥30 kg/m^2^ was comparable to values reported previously for the prevalence of obesity in the same geographic region [42].

In summary, although Bergman et al. found that the BAI is a good tool to estimate adiposity in Caucasian populations and suggested that it is more practical and easier than other complex mechanical systems, results from the present study suggest that the BAI does not overcome the limitations of BMI and also it is not a good tool to measure metabolic and cardiovascular health risk in Caucasian Populations. Thus, BAI is less useful not only than BMI but also than other adiposity indexes such as WHtR, WHR and WC. These adiposity indexes may be better candidates for use in clinical practice and research to evaluate both metabolic and cardiovascular risk.
